# Comparative Study of Selenides and Tellurides of Transition Metals (Nb and Ta) with Respect to its Catalytic, Antimicrobial, and Molecular Docking Performance

**DOI:** 10.1186/s11671-020-03375-0

**Published:** 2020-07-08

**Authors:** S. Altaf, A. Haider, S. Naz, A. Ul-Hamid, J. Haider, M. Imran, A. Shahzadi, M. Naz, H. Ajaz, M. Ikram

**Affiliations:** 1 Department of Chemistry, University of Engineering and Technology, Lahore, 54000 Pakistan; 2 Department of Clinical Medicine and Surgery, University of Veterinary and Animal Sciences, Lahore, Punjab 54000 Pakistan; 3 Tianjin Institute of Industrial Biotechnology, Chinese Academy of Sciences, Tianjin, 300308 China; 4 Center for Engineering Research, Research Institute, King Fahd University of Petroleum & Minerals, Dhahran, 31261 Saudi Arabia; 5 State key Laboratory of Chemical Resource Engineering, Beijing Advanced Innovation Centre for Soft Matter Science and Engineering, Beijing Engineering Center for Hierarchical Catalysts, Beijing University of Chemical Technology, Beijing, 100029 China; 6 University College of Pharmacy, University of the Punjab, Lahore, 54000 Pakistan; 7 Biochemistry Lab, Department of Chemistry, Government College University, Lahore, Punjab 54000 Pakistan; 8 Solar Cell Applications Research Lab, Department of Physics, Government College University, Lahore, Punjab 54000 Pakistan

**Keywords:** Selenides, Tellurides, Solid-state synthesis, Catalysis, Antimicrobial, Molecular docking

## Abstract

The present research is a comparative study that reports an economical and accessible method to synthesize niobium (Nb) and Tantalum (Ta) selenides and tellurides with useful application in the removal of pollutants in textile, paper, and dyeing industries as well as in medical field. In this study, solid-state process was used to generate nanocomposites and various characterization techniques were employed to compare two groups of materials under investigation. Structure, morphology, elemental constitution, and functional groups of synthesized materials were analyzed with XRD, FESEM coupled with EDS, FTIR, and Raman spectroscopy, respectively. HR-TEM images displayed nanoscale particles with tetragonal and monoclinic crystal structures. The optical properties were evaluated in terms of cut-off wavelength and optical band gap using UV-visible spectroscopy. A comparative behavior of both groups of compounds was assessed with regards to their catalytic and microcidal properties. Extracted nanocomposites when used as catalysts, though isomorphs of each other, showed markedly different behavior in catalytic degradation of MB dye in the presence of NaBH_4_ that was employed as a reducing agent. This peculiar deviation might be attributed to slight structural differences between them. *Escherichia coli* and *Staphylococcus aureus* (G –ve and + ve bacteria, respectively) were designated as model strains for in vitro antibacterial tests of both clusters by employing disk diffusion method. Superior antibacterial efficacy was observed for telluride system (significant inhibition zones of 26-35 mm) compared with selenide system (diameter of inhibition zone ranged from 0.8 mm to 1.9 mm). In addition, molecular docking study was undertaken to ascertain the binding interaction pattern between NPs and active sites in targeted cell protein. The findings were in agreement with antimicrobial test results suggesting NbTe_4_ to be the best inhibitor against FabH and FabI enzymes.

## Introduction

Transition metal chalcogenides TMCs (where M = Ti, V, Nb, Ta, Mo, W, etc.; C = S, Se, Te) are highly promising materials that are suitable for use in a number of industrial sectors including electronics, energy conversion and storage, photovoltaic, thermoelectric, and catalysis [1, 2] owing to their desirable optical, electrical, and electrochemical properties [3]. Recently, selenides and tellurides doped with niobium and tantalum have received significant attention due to its potential use in applications such as in semiconductors, upper conversion of IR to visible light [4, 5], gas sensors [6], laser diodes, medical diagnostics, photodetection devices, photocatalysis [7], superconductors, and topological insulators or semimetals [8]. Generally, transition metal chalcogenides exist as MC_2_, MC_3_, and MC_4_ systems where M is known as a transition metal and C = S, Se, or Te [9]. The lower selenides and tellurides, MC_2_, have highly layered two-dimensional (2D) structure [10] with metal atom located between the layers at octahedral sites [11]. Two dimensional (2D) materials including transition metal dichalcogenides, graphene (first 2D material discovered in 2004) [12], black phosphorus, and hexagonal boron nitride [13] have been extensively explored due to their unique electronic, structural, optical, and magnetic properties [14]. On the other hand, MC_3_ and MC_4_ are apparently non-layered structures which crystallize in quasi-one-dimensional configurations [15, 16] with infinite chains of MC. Although, chemical formula appears to be similar for both families but structurally they are slightly different from each other. These structural differences give rise to variations in their electrical transport properties [17–19]. An important feature of tellurides that distinguishes it from the sulfides and selenides in its crystal structure, electronic configuration, and physical properties is the large atomic number of Te. The diffusive character of valence orbitals [20] of Te and its more covalent nature [21] results in strong spin-orbital (SO) coupling [22]. Presently, materials with strong SO coupling are of great interest in condensed matter physics [23]. In this regard, superconductivity in low-dimensional quasi (1D) tellurides (NbTe_4_) with a large atomic number is under investigation [21, 24]. To meet the increasing demand of nanostructured selenides and tellurides of TM in various fields, a number of approaches have been adopted to synthesize these materials including sol-gel [7, 25, 26], electrospinning [27], oriented-attachment process [28, 29], chemical vapor deposition [30], organic solution-based high-temperature synthesis [31], template-directed method [32], and hydrothermal/solvothermal reaction [33].

Substantial environmental pollution caused by toxicity of organic dyes and pigments discharged as effluents from various industries remains a major source of health risk at a global level. It is not feasible to eliminate these non-biodegradable wastes through conventional water treatment methods [7, 34] due to their complex aromatic structures, hydrophilicity, and stability against light, chemicals, and temperature [35, 36]. Therefore, the development of effective, convenient, and economical degradation techniques has received paramount attention recently [37]. To date, various practices based on physical, biological, and chemical methods have been adopted to treat wastewater polluted with dyes [38]. These methods lack practical utility due to high capital cost, low efficiency, sluggishness, and high energy input. On the contrary, catalytic reduction process is a preferred option since it is relatively quick, inexpensive, and low-temperature treatment [35, 37]. In this regard, several transition metal chalcogenides such as VSe_2_ (photocatalyst and supercapacitor) [39], Yb-doped WTe_3_ (ultra-short laser and amplifier) [40, 41], TaSe_3_ (superconductor), TaS and NbSe_3_ (semiconductors) have been reported in the literature [7, 27]. Here, it is pertinent to point out that much less attention has been paid to the study of catalytic reduction of methylene blue (MB) with NaBH_4_ by utilizing compounds studied here.

In the biomedical field, antimicrobial properties of metallic composites have long been recognized and successfully used for some of the most extraordinary innovations in the history of medicine [42, 43]. Among transition metals, Ta compounds are reported as good antimicrobial agents due to them being unreactive, nontoxic, and biocompatible. Whereas, researches on Nb composites used as biocidal agents are fairly limited [44].

In view of above, we intended to synthesize selenides and tellurides of transition metals (Nb, Ta) and to undertake a comparative study by evaluating their catalytic and antimicrobial properties. To the best of our knowledge, such a comparative investigation has not been reported to date. Solid-state technique was used to synthesize selenides (TaSe_3_, Nb_2_Se_3_) and tellurides (TaTe_4_, NbTe_4_) and isolated products were characterized via elemental analysis, FTIR, Raman, EDS, FESEM, HRTEM, and UV-Vis spectroscopy. Furthermore, molecular docking study was carried out to evaluate the binding interaction pattern of NPs with cell proteins of locally isolated bacterial strains including *E. coli* and *S. aureus*. The aim of the present study was to assess the comparative behavior of tellurides and selenides of transition metals with regards to their catalytic, microcidal, and molecular docking performance.

## Methods

The current study aimed a comparative behavior of tellurides and selenides of transition metals was assessed to investigate their catalytic, microcidal properties, and molecular docking analysis.

### Chemicals

Niobium pentachloride-NbCl_5_ (99%), tantalum pentachloride-TaCl_5_ (99.8%), selenium dioxide-SeO_2_ (≥ 99.9%), tellurium dioxide-TeO_2_ (≥ 99%), methylene blue (MB), sodium borohydride (NaBH_4_), and nitric acid HNO_3_ (65%), were obtained from Sigma-Aldrich. Hydrochloric acid-HCl (37%) was acquired from Riedel-de Haen. Aqua regia was used to wash glassware followed by rinsing with double-distilled water twice. Nutrient agar was purchased from Merck. Pure cultures of bacterial strains were provided by the Zoology Department, G.C. University, Lahore. Additional purification treatment was not undertaken prior to use.

### Synthesis of Transition Metal Selenides and Tellurides

**Fig. 1:**
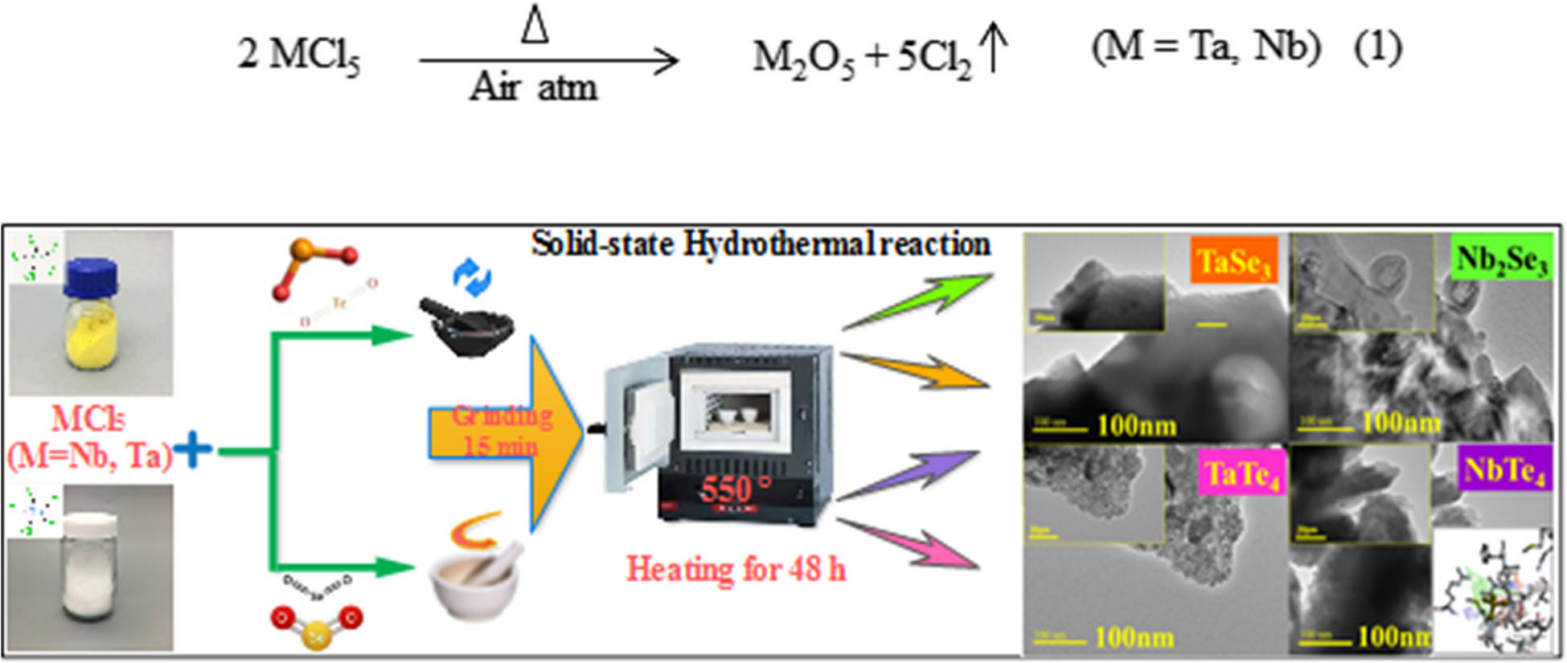
Schematic diagram of synthesis of metal selenides and tellurides

Two groups namely tellurides and selenides of transition metals (Ta & Nb) with compositions of (TaTe_4_, NbTe_4_) and (TaSe_3_, Nb_2_Se_3_), respectively were synthesized via standard solid-state technique (see Fig. 1). Precursor metal chlorides (TaCl_5_, m.p: 216 °C and NbCl_5_, m.p: 204.7 °C) each were thoroughly mixed and milled with chalcogen oxides (TeO_2_, m.p: 732 °C and SeO_2_, m.p: 340 °C) for 15 min to enhance the contact area between particles and ensure homogeneity. The resultant mixture was melted by placing it in a muffle furnace held at 550 °C for 48 h. Initially, the temperature of the furnace was raised at the rate of 50 °C/h. Afterwards, the furnace was cooled at the rate of 10 °C/h to room temperature and the products were extracted. Chlorine in the precursor metal chloride oxidizes when subjected to high temperature, as shown by the following possible mechanism [45].

### Catalysis

The catalytic potential of synthesized nanocomposite was evaluated by measuring the reduction rate of methylene blue-MB in the presence of NaBH_4_. MB is a cationic dye, widely used in chemistry labs as a redox indicator. One millimolar of MB solution was diluted with deionized water followed by the addition of 400 μl freshly prepared sodium borohydride-NaBH_4_ solution (100 mM) in it. Later, synthesized compounds were incorporated into the solution and stirred for 5 min. Decolorization of sample represents the conversion of MB to leucomethylene blue (LMB) in the presence of sodium borohydride (see equation 2). The reaction was monitored spectrophotometrically and absorption at 665 nm was recorded at regular time intervals at 25 °C in the range of 250–750 nm.

### Antimicrobial Activity

Bactericidal action of synthesized material was studied through disk diffusion assay against gram-positive (G + ve) *Staphylococcus aureus* and gram-negative (G –ve) *Escherichia coli* using Whatman filter papers as disk under aseptic conditions. Bacterial cultures containing 1 × 10^7^ CFU/mL were spread on nutrient agar plates while various concentrations (0.25, 0.5, and 1 mg/ml) were utilized to check synthesized nanocomposites susceptibility in comparison with deionized water (DIW) as negative control. The inoculated agar plates were incubated aerobically overnight at 37 °C and inhibition zones were measured using meter scale (mm) (Image J software). Results reproducibility as well as reliability was ensured by repeating experiment in thrice.

### Materials Characterization

Information about the structure and crystal phases of the synthesized products was obtained with X-ray diffractometer (model: PANalytical X’Pert PRO) operating at 40 kV and 30 mA using Cu-Kα radiation (*λ* = 1.540 Å) with 2θ variation from 20-80° at a scanning rate of 0.02°/s. The Philips proprietary software, X’Pert high score plus was used for curve fitting and integration. The morphological properties and elemental composition were attained via (JSM-6460LV) FESEM equipped with an energy dispersive X-ray EDS spectrometer. Moreover, inter-layer spacing was assessed with the help of HRTEM of model Philips (CM30) and JEOL (JEM 2100F). Optical characteristics were determined using UV-Vis (GENESYS 10S) spectrophotometer operated at a range from 120 to 1100 nm. FTIR was engaged to detect the functional groups through Perkin Elmer spectrometer used in the range of 4000-400 cm^−1^. Raman scattering experiments were conducted on powdered samples with Raman spectrometer fitted with diode laser as an excitation source focused at a wavelength of 532 nm.

### Molecular Docking Study

Molecular docking study of synthesized tellurides and selenides was performed to understand the mechanism underlying bactericidal activity. This was undertaken by targeting proteins crucial for bacterial survival and growth. Multiple protein targets belonging to various biosynthetic pathways were selected for molecular docking study namely, *β*-lactamase, dihydrofolate reductase, enoyl-[acyl-carrier-protein] reductase (FabI), and beta-ketoacyl-acyl carrier protein synthase III (FabH). The *β*-lactamase and dihydrofolate reductase play pivotal role in the biosynthesis of the cell wall and folic acid, respectively which is needed for bacterial survival. Similarly, FabH and FabI enzymes catalyze key steps in the fatty acid biosynthetic pathway of bacterial cell [46–48].

**Fig. 2:**
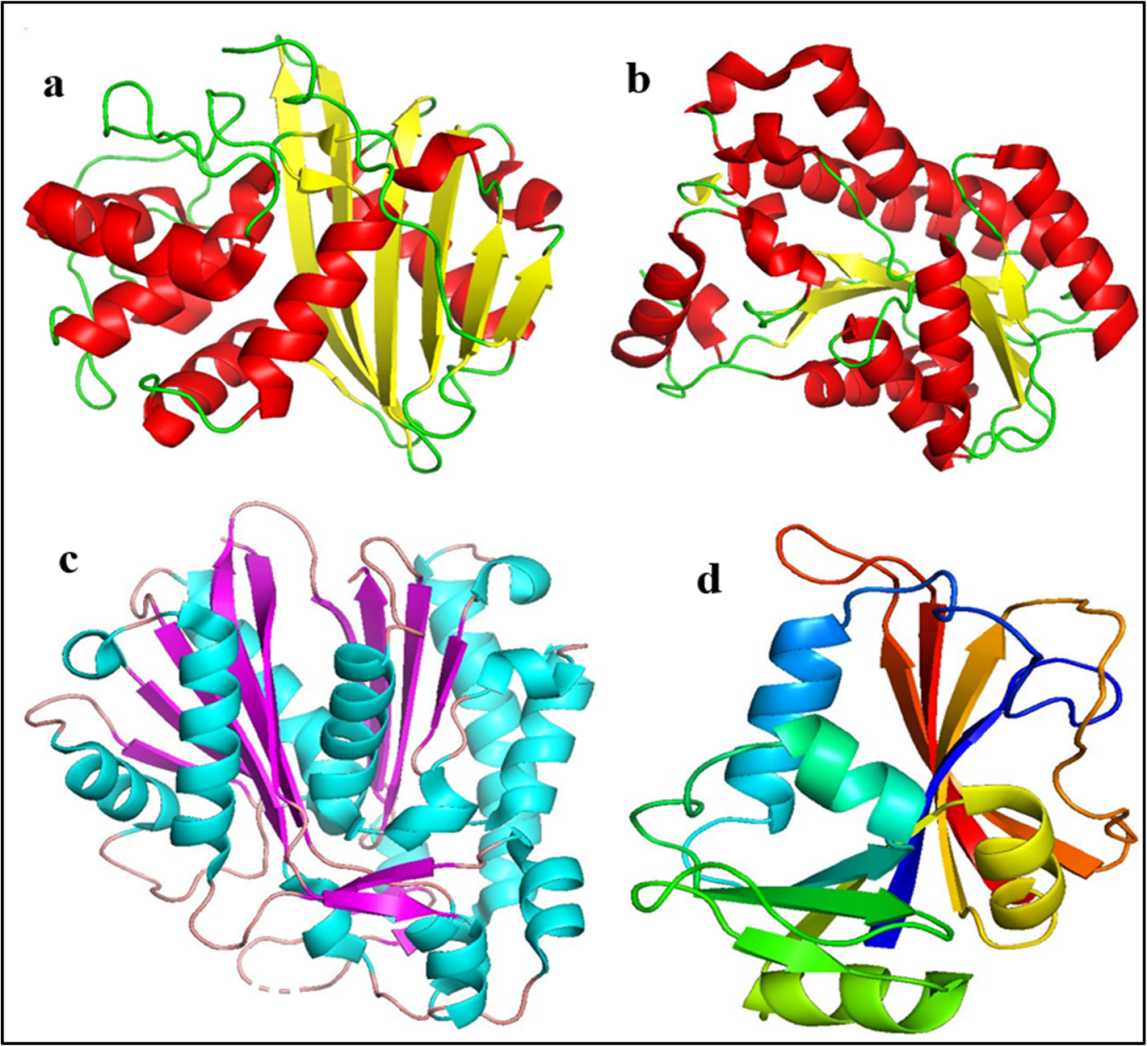
3D-structure of target proteins. **a** Beta lactamase (PDB: 3Q81; *S. aureus*). **b** FabI (PDB: 4D41; *S. aureus*). **c** FabH (PDB: 5BNR; *E. coli*). **d** DHFR (PDB: 1RD7; *E. coli*)

High quality crystallographic structures of target proteins of E.coli and S. aureus with good resolution were retrieved from Protein data bank (Fig. 2). The proteins with PDB ID: 3Q81; Resolution: 2.1 Å [49], 1RD7; Resolution: 2.6 Å [50], 4D41; Resolution 2.3 Å [51], 5BNR; Resolution: 1.9 Å [52] were selected to understand molecular interactions between nanoparticles and active pocket residues of protein.

The ICM v3.8-4a or above (Molsoft L.L.C., La Jolla, CA) software was employed for molecular docking study [53]. The receptor preparation tool of ICM Molsoft was employed for protein structure preparation that involved ***the*** removal of water molecules and co-crystallized ligand. In addition, energy minimization and optimization of protein structures was undertaken using default parameters and force field. Later, binding pocket was specified using ***a*** grid box and 10 best***-***docked conformations were generated to examine the interaction pattern of nanoparticles with active site residues.

Previously reported structure of tellurides and selenides were retrieved from PubChem in .cif format and used for the preparation of Ta-doped, Nb-doped tellurides***,*** and selenides structure using ***the*** Gaussian 09 software and ligand preparation tool of ICM Molsoft.

## Results and Discussion

**Fig. 3:**
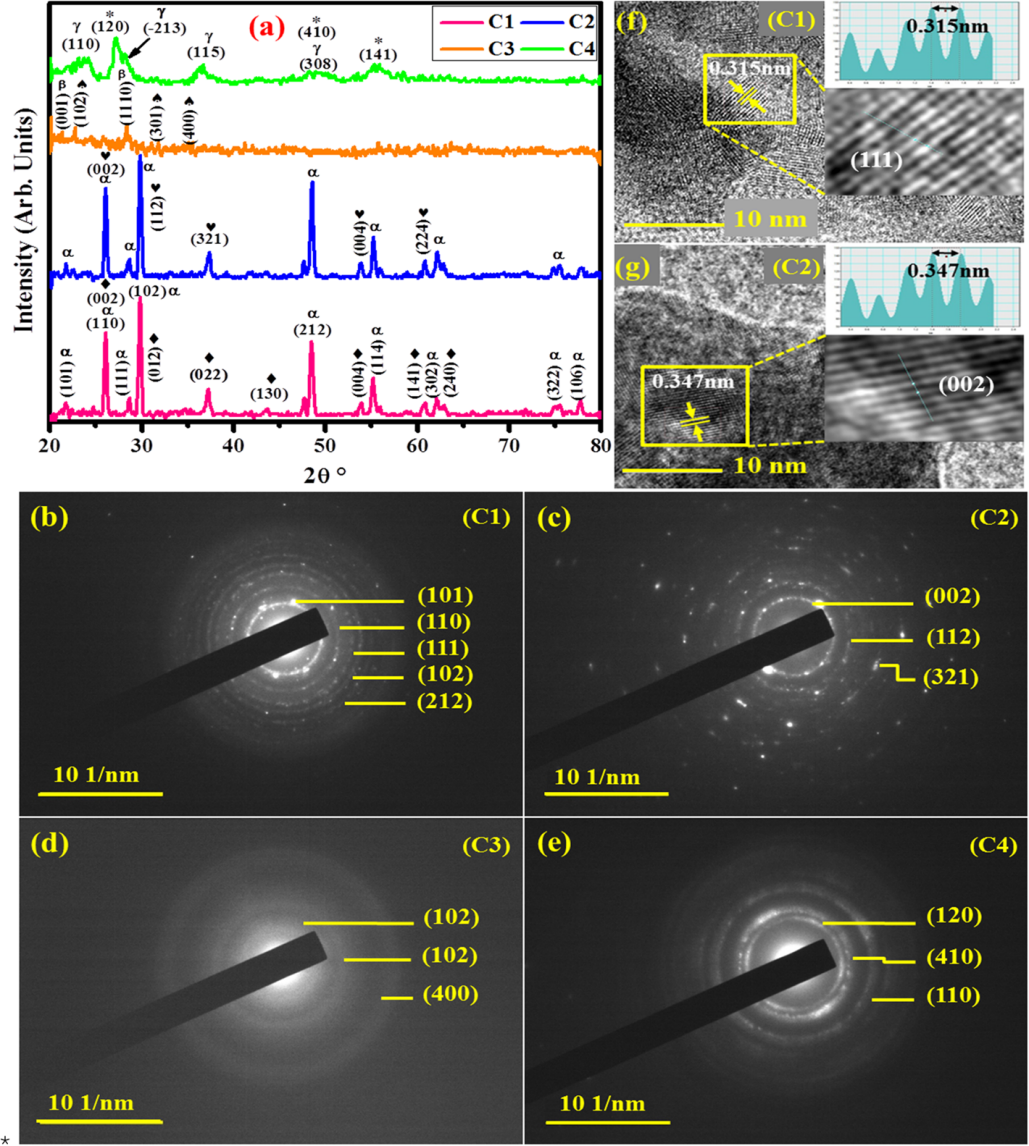
**a** XRD patterns. **b-e** SAED images of C1, C2, C3, and C4 samples (550 **°**C) for 48 h. **f-g** HRTEM micrograph of nanocrystals (C1 & C2) showing lattice-fringed spacing

Figure 3a depicts XRD patterns of prepared composites after annealing at 550 °C. Characteristic peaks of all samples were closely matched with the database of JCPDS. In telluride group, tetragonal structures of TaTe_4_ (♦) in C1 (by Brandon and Lessard 1983) [16, 54] and NbTe_4_ (♥) in C2 (JCPDS 77-2283) [55] were major phases identified in XRD patterns. The common diffraction peaks (marked as **α**) at 21.8° (101), 26.1° (110), 28.6° (111), 29.8° (102), 48.4° (212), 55.1° (114), 62.2° (302), 75.1° (322), and 77.7° (106) can be indexed to unreacted tetragonal crystal structure of TeO_2_ (m.p: 732 °C) (JCPDS card No.78-1713) [56]. In case of selenide group, C3 and C4, monoclinic phases of TaSe_3_ (♠) JCPDS file: 18-1310 [7, 57] and Nb_2_Se_3_ (*) JCPDS card no. 01-089-2335 [1], respectively were detected and assigned to hkl planes. Moreover, respective transition metal oxides were also formed in as-prepared samples. In C3 diffraction peaks (**β**) at 2θ = 22.8° and 28.4° corresponding to (001) and (1110) planes were ascribed to orthorhombic Ta_2_O_5_ phase according to (JCPDS 025-0922) [45]. Peaks shown by C4 located at 23.7 (110), 27.2 (−213), 36.7 (115), and 50.1 (308) can be attributed to monoclinic Nb_2_O_5_ (**γ**) as reported in (JCPDS file No. 37-1468) [58]. Average crystallite size of C1, C2, C3, and C4 nanoparticles (22.2, 22.16, 26.7, and 10.04 nm respectively) was calculated using FWHM according to Debye-Scherrer formula. Additional confirmation of the crystalline texture of grown nanoparticles was achieved by using selected area electron diffraction (SAED) patterns of HR-TEM. Both diffraction techniques SAED and XRD are analogs of each other; however, the former differ only in respect of using an electron beam instead of monochromatic X-rays [59]. XRD is the primary technique used for the identification of crystal structure but can barely be used for heterogeneous nanocrystalline samples. It is capable of detecting electron density distribution only due to relatively weak interaction of X-rays with electrons alone while in high-resolution TEM, electron beam strongly interacts both with electric as well as nuclear field, thus giving highly magnified crystal structure as compared to that of X-ray diffraction [59, 60]. Figure 3b-e demonstrates the (SAED) patterns of corresponding samples with concentric rings indexed to hkl reflection planes which are consistent with XRD results [61, 62]. The bright reflection spots in several concentric circles in SAED patterns (b and c) indicated that synthesized nanocomposites were crystalline while weak reflection observed among these bright rings revealed the presence of an amorphous compound. Moreover, the absence of bright spots in SAED rings of (d) and few spots in (e) depicts the amorphous and slight crystalline nature of these nanostructures, respectively [63, 64]. For further insight into the crystal structure, HRTEM of C1 and C2 was carried out and outcomes are presented in Fig. 3f, g. The clear lattice fringes in HRTEM images indicate the high crystallinity of nanoparticles [65] with d-spacing of ~ 0.315 and 0.347 nm that corresponds well with the interplanar distance of (111) plane of tetragonal TeO_2_ (JCPDS no. 78-1713), and (002) plane of tetragonal NbTe_4_ (JCPDS 77-2283) [55], respectively.

**Fig. 4:**
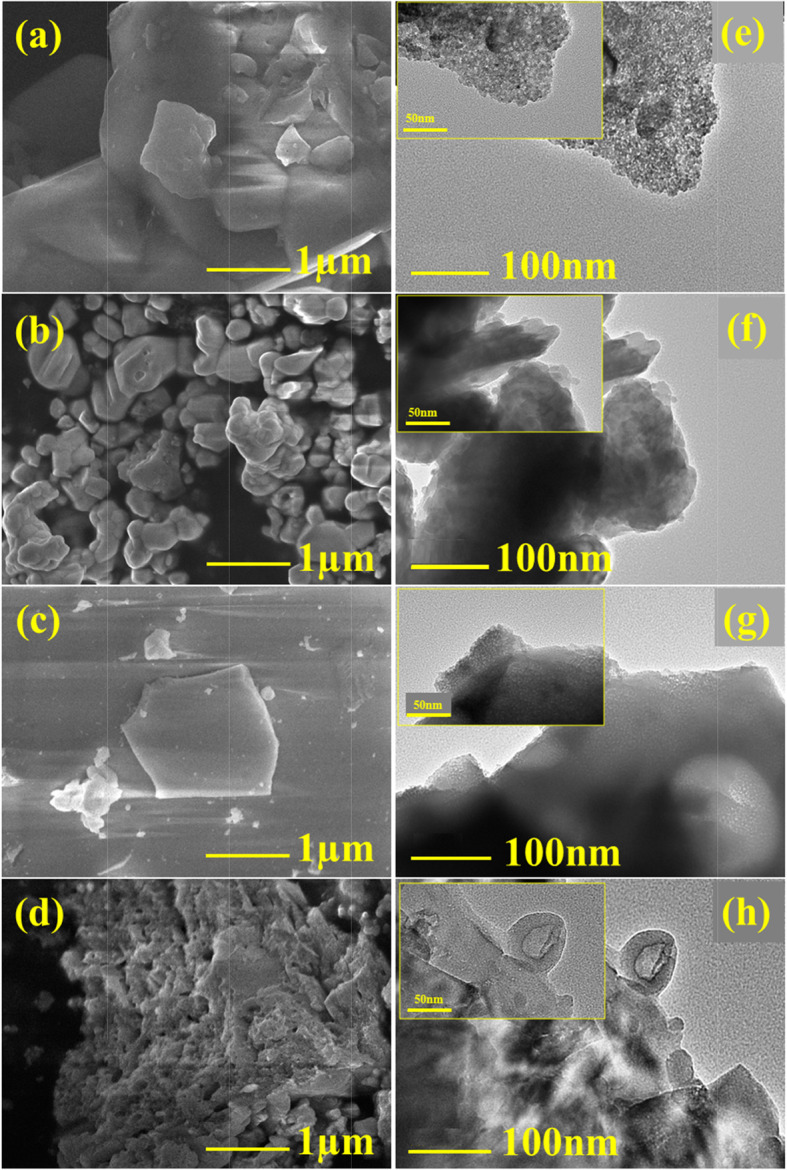
**a-d** FESEM in micrometer scale. **e-h** Low magnification HRTEM micrographs of C1, C2, C3, and C4 NPs with high magnification inset

Representative FESEM and HRTEM images of final products synthesized after annealing at 550 °C for 48 h, are shown in Fig. 4 for further elaboration of surface morphology and crystal structure of NPs. FESEM micrographs of telluride group (a and b) possess flake-like microcrystals in C1 while non-uniform irregular shaped particles with a tendency to aggregate with an average diameter of 22 nm are observed in C2. FESEM images of selenide group (c and d) indicate the plate-/disk-like structures dispersed over a flat surface (C3) and particle agglomeration can be observed in C4 morphology with the size of NPs ranging from 10 to 27 nm. These structures become more evident when examined with high-resolution TEM at higher magnification (see Fig. 4e-h) and insets (top left with 50 nm resolution) also confirm the formation of nanoparticles.

**Fig. 5:**
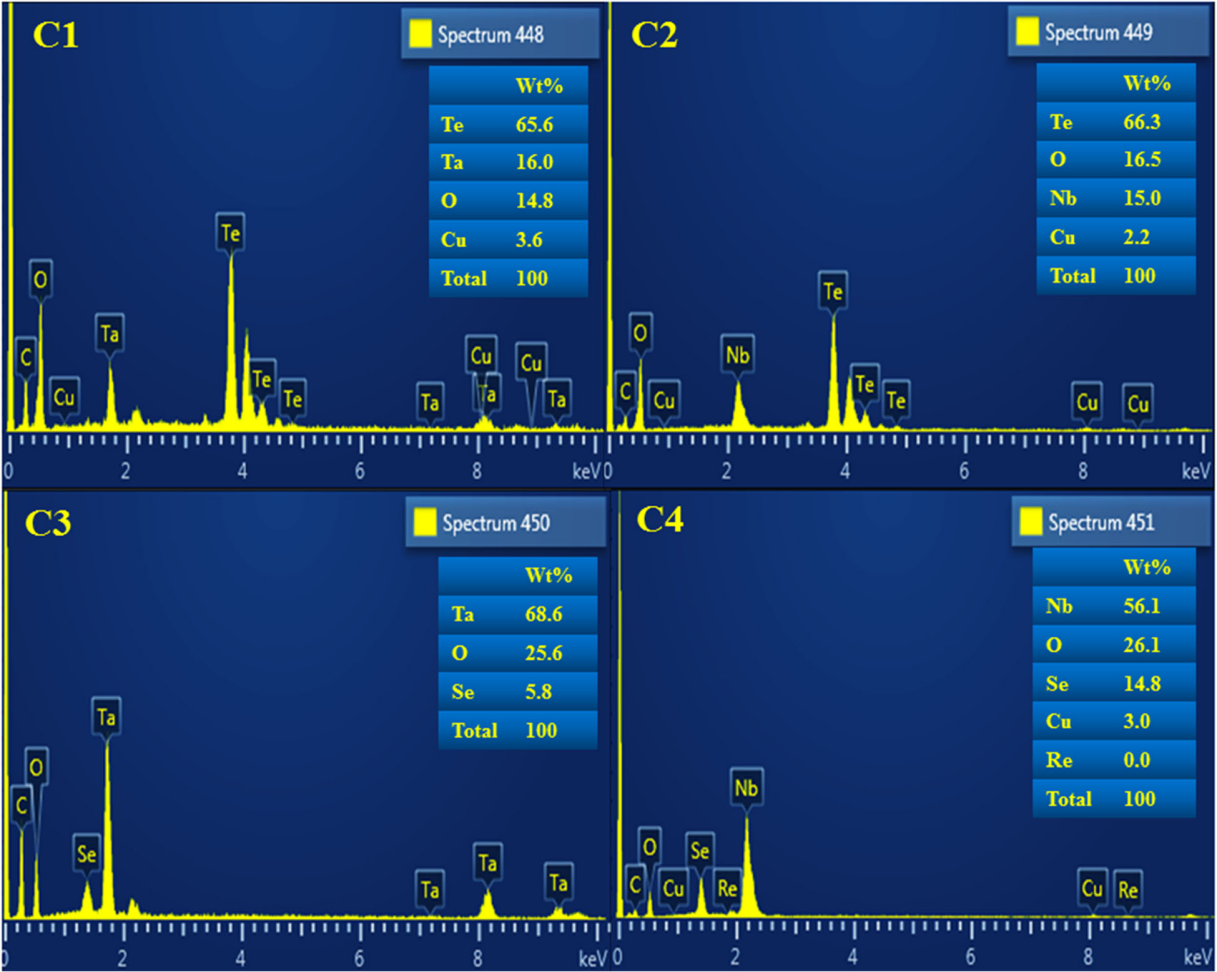
EDS spectra of C1, C2, C3, and C4 samples

Energy dispersive X-ray spectroscopy (EDS) was utilized to determine the elemental composition of synthesized nanocomposites. EDS spectra (Fig. 5) confirmed the presence of all constituent elements (Ta, Nb, Te, Se, and O) in respective samples. From spectra, it is shown that the prepared samples possess 16.0 wt% of Ta and 65.6 wt% of Te in C1 and 15 wt% of Nb and 66.3 wt% of Te in C2, these values are consistent with the relative atomic ratios of elements (~ 1:4) present in compounds TaTe_4_ and NbTe_4_, respectively. Carbon and copper signals arise from the carbon-coated samples and Cu grids used for FESEM measurements [7, 66]. No other peaks or elements associated to contaminations were observed in spectra which assure the purity of consequent products formed as a result of solid-state synthesis.

**Fig. 6:**
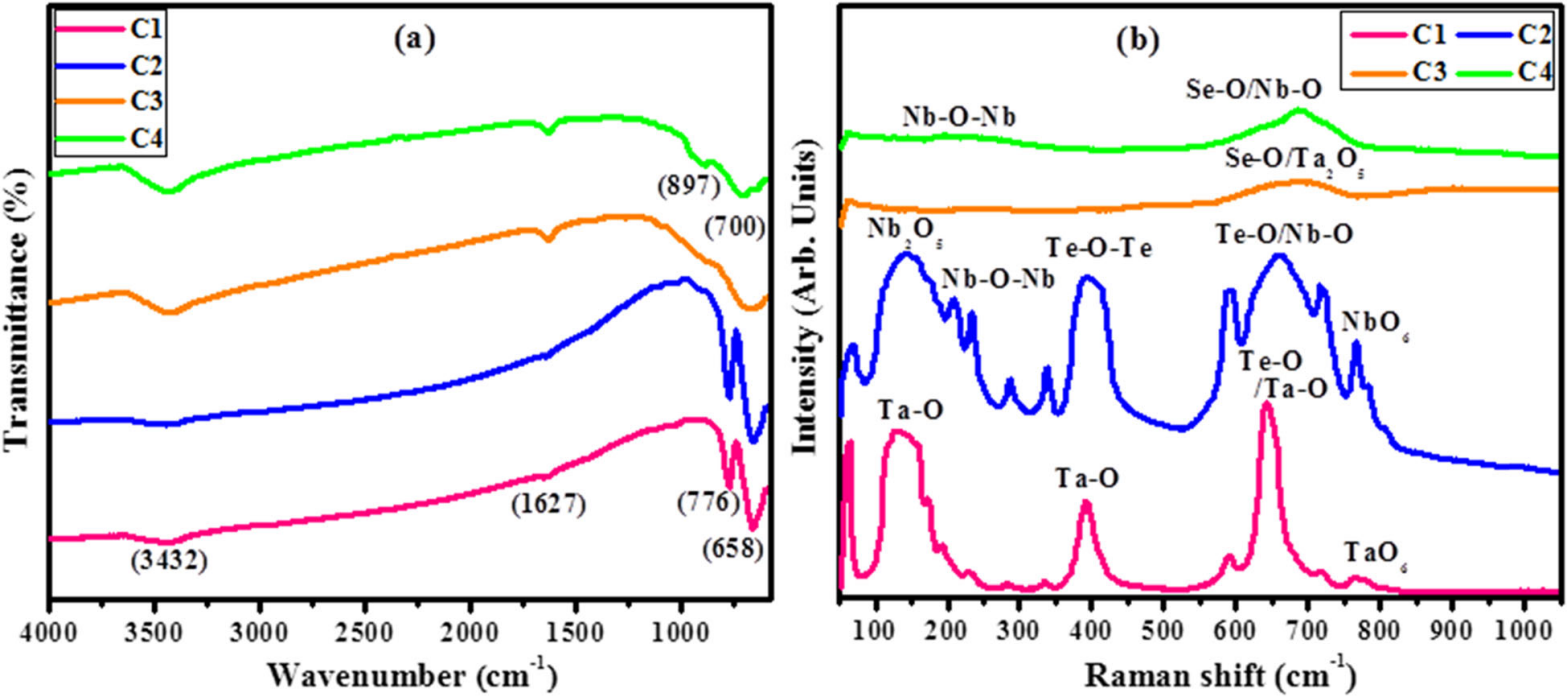
**a** FTIR spectra. **b** Raman spectra of C1, C2, C3, and C4

The nature of chemical bonding and functional groups of synthesized composites C1, C2, C3, and C4 was elucidated through FTIR studies conducted in the range of 400-4000 cm^−1^ (Fig. 6a). Significant transmission bands observed at 3432 and 1627 cm^−1^ are associated with the stretching frequency of O-H group [7]. In tellurides spectra C1 and C2, the observed intense peaks at 658 cm^−1^ correspond to the stretching vibrations of Te-O bonds in trigonal bipyramidal (tbp) TeO_4_ units [67] while the vibrational band at 776 cm^−1^ reveals the existence of Ta-O-Ta bond in C1 [68] and NbO_4_ tetrahedral unit in C2 [69]. Spectra of prepared selenide composites C3 and C4 indicate the presence of Se-O bonds due to stretching vibrational modes near 700 cm^−1^ [70] Peaks transmitted in the region of 700-900 cm^−1^ are assigned to the metal oxide bond as Ta-O-Ta bond [3] in C3 and Nb-O bond [69] in C4. Raman spectroscopy was undertaken in the range of 50-1050 cm^−1^ to disclose various structural units of synthesized composites (Fig. 6b). Raman scattering in telluride samples (C1, C2) was observed in three regions including 100-250, 350-450, and 550-850 cm^−1^. First region at 100-250 cm^−1^ corresponds to stretching vibration of Ta-O unit in C1, vibrational bending of Nb-O-Nb linkage, and Nb_2_O_5_ octahedron in C2 [71–74]. Promising peak in second region 350-450 cm^−1^ is attributed to symmetric stretching of Te-O-Te bonds [75]. The third section comprised of a broad band located at 550-850 cm^−1^ and is symmetric to Raman modes for terminal Ta-O bond, Te-O/Nb-O stretching vibrations, and TaO_6_/Nb_2_O_5_ octahedral modes in C1 and C2, respectively [71–74]. In case of selenide compounds, C3 and C4 (samples are amorphous or possess low degree of crystallinity as observed in XRD and HRTEM results) Raman shifts are observed only between 580-780 cm^−1^, which indicates the presence of Ta_2_O_5_ moiety in C30 [68] and the stretching of Nb-O bond in C4 [72, 73] in addition to terminal Se-O bridging vibrations [76]. The observed Raman scattering and vibrational modes observed in the spectra of synthesized nanocomposites correlate with the structural relationship derived from XRD data.

**Fig. 7:**
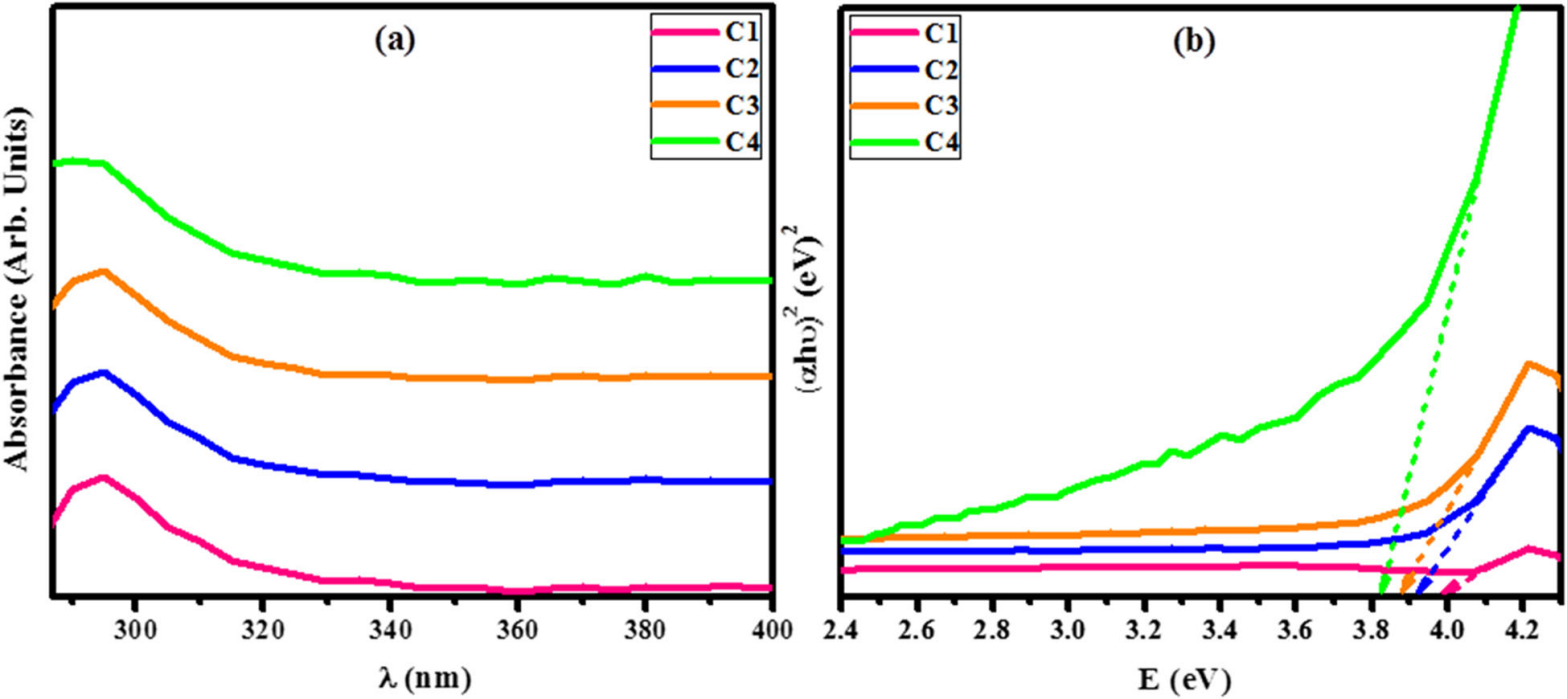
**a** UV-Vis spectra. **b** Band gap of C1, C2, C3, and C4

Optical measurements and additional structural properties of as-prepared samples C1, C2, C3, and C4 were carried out in the range of 285-400 nm by employing UV-Vis (GENESYS 10S) spectrophotometer. Figure 7a illustrates the absorption curves plotted against absorbance along ordinate and wavelength (nm) along the abscissa. All the grown samples showed absorbance in the UV zone as depicted by absorption curves in the range of 250-350 nm [77, 78]. An intense absorption band was observed immediately below the absorption edge near 292 nm both in tellurides and selenides due to electronic transitions in Ta^+5^, Nb^+5^ ions, and/or a lone pair of electrons on Te/Se atoms. The absorption cut-off wavelength is taken at where an abrupt increase in optical absorption starts [4] and it is the wavelength, which is used to evaluate the optical band gap of composites [79, 80]. The direct band gap energies (Eg) were estimated by plotting (*αhυ*)^2^ along *y*-axis and *hυ* on *x*-axis followed by extrapolating the linear fits to the *x*-axis (Fig. 7b). The intercept values on *x*-axis (3.99, 3.91, 3.87, and 3.82 eV) correspond to the estimated band gaps of C1, C2, C3, and C4 respectively, which indicates that the subsequent NPs are wide band gap materials.

**Fig. 8:**
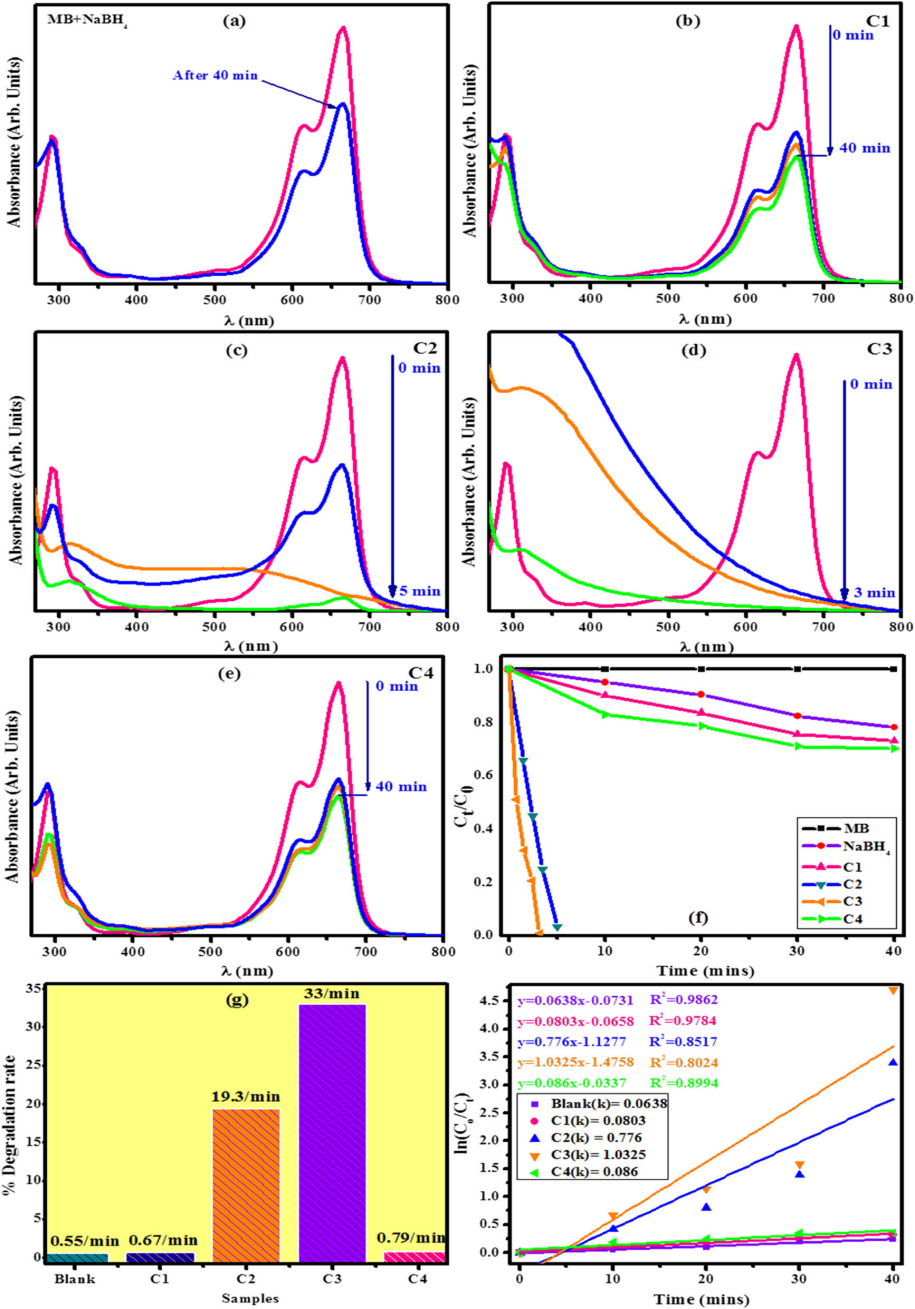
Time dependent UV–Vis spectra for the reduction of MB. **a** MB with NaBH_4_. **b** MB with NaBH_4_ + C1. **c** MB with NaBH_4_ + C2. **d** MB with NaBH_4_ + C3. **e** MB with NaBH_4_ + C4

Figure 8a-e illustrates the catalytic degradation of methylene blue in the presence of NaBH_4_ and nanocomposites examined using a spectrophotometer. Degradation rate of dye in the presence of sodium borohydride is shown in Fig. 8a, while catalytic efficiency of TaTe_4_ (C1), NbTe_4_ (C2), TaSe_3_ (C3), and Nb_2_Se_3_ (C4) is illustrated in Fig. 8b-e. Significant catalytic activity was recorded for samples C2 and C3, as these results in successive decrease in the concentration of methylene blue. It took merely 3-5 min for the conversion of MB to leucomethylene blue as shown in Fig. 8c, d. Negligible catalytic performance was noted for samples C1and C4 as shown in Fig. 8b, e. Low catalytic efficiency might be explained due to slight structural differences that exist in the materials’ quasi-dimensional arrangement of MC chains despite possessing chemically isomorph configurations [17–19]. Dye degradation curves of synthesized nanocomposites are shown in Fig. 8f. Degradation curves of sample C2 and C3 represent sharp decline, while curves of other samples show a slight difference from standard MB curve, which demonstrates that samples C2 and C3 possess higher catalytic potential as compared to the rest of the samples. The % degradation of methylene blue was calculated by using the following equation:

% Degradation = 100 − (*C*_**t**_ × 100∕*C*_**o**_) (3)

where initial absorbance of MB is represented as *C*_**o**_ and absorbance at time *t* is shown as *C*_***t***_. Significant dye degradation potential was shown by samples C2 (19.9%/min) and C3 (33%/min); However, negligible activity was observed in the remaining samples as shown in Fig. 8g. The pseudo-first-order reaction [81] was used to estimate the catalytic efficiency of synthesized nanocomposite quantitatively. Following expression (equation 4) was employed to calculate the rate constant.

ln [*C*_o_∕*C*_t_] = *kt* (4)

Here, *C*_o_ is the initial concentration of the dye and *k* represents rate constant [82]. Figure 8h illustrates the rate constant values obtained from the absorbance curve. These values are 1.0325/min, 0.776/min, 0.086/min, and 0.0803/min for C3, C2, C4, and C1, respectively. Sample C3, with a high rate constant, exhibits significant catalytic proficiency for methylene blue degradation. Similarly, C2 also depicts high catalytic efficiency while remaining samples are not proved as effective nanocatalysts.

**Fig. 9:**
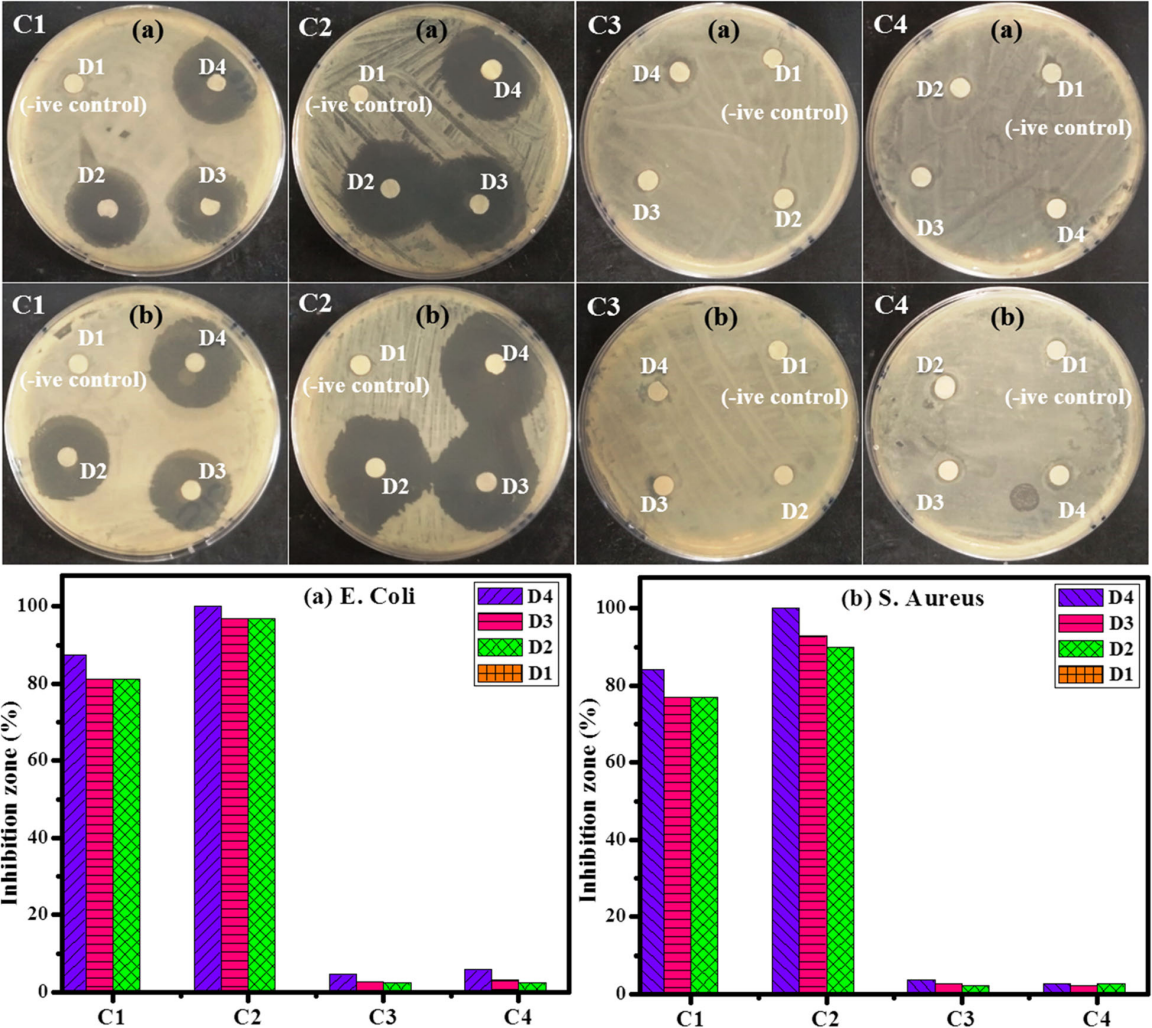
Disk diffusion assay and qualitative antibacterial assessment of C1, C2, C3 and C4 against (**a**) Escherichia coli and (**b**) Staphylococcus aureus

Agar disk diffusion assay was used to assess the antibacterial sensitivity of the prepared tellurides (C1, C2) and selenides (C3, C4) of Nb and Ta. Zones of inhibitions were recorded for samples against *E. coli* and *S. aureus*as as shown in Table 1. Antibacterial activity of the tellurite group is greater than C3 and C4 (Fig. 9); although, the maximum inhibitory zone was recorded for sample C2 at a concentration of 1 mg/ml for *E. coli* (35 mm) and *S. aureus* (32 mm). Similarly, sample C1 also showed maximum inhibition at D4 concentration, i.e., 28 and 29.5 mm for *E. coli* and *S. aureus*, respectively. However, D2 and D3 concentrations showed lower bactericidal activity than D4; this trend represents the dose-dependent cytotoxic effect of nanocomposites. Direct proportionality was observed between the synergistic effect and the NPs concentrations and inhibition zones (mm) [83]. Bar graph represents negligible bactericidal activity for sample C3 (TaSe_3_) and C4 (Nb_2_Se_3_) due to the presence of selenium (Se) since it is an essential micronutrient that enhances bacterial growth and decreases the antibacterial potential of samples [84]. The marked decrease in bacterial growth in case of tellurites with an increase in nanoparticles concentration is attributed to the formation of reactive oxygen species (ROS) that causes oxidative stress as a result of the redox reaction of metal ions, which inhibits the growth of particular enzymes and destroys the bacterial DNA leading to the death of bacteria [85]. The overall charge on harvested composites was positive while the bacterial cell wall is anionic in nature. One possible reaction mechanism could be the cationic interaction of metal ions, which renders bacterial ribosomes and enzymes dysfunctional, consequently resulting in the collapse of micro-pathogens [86].

**Table 1:** Antimicrobial activity of C1, C2, C3, and C4

Micro organism	Sample	Inhibition zone (mm)			
		D4 (1 mg/ml)	D3 (0.5 mg/ml)	D2 (0.25 mg/ml)	D1 (DIW)
a) *E. coli*	C1	28	26	26	0.0
	C2	32	31	31	0.0
	C3	1.5	0.9	0.8	0.0
	C4	1.9	1.0	0.8	0.0
b) *S. aureus*	C1	29.5	27	27	0.0
	C2	35	32.5	31.5	0.0
	C3	1.3	1.0	0.8	0.0
	C4	1.0	0.8	0.9	0.0

**Fig. 10:**
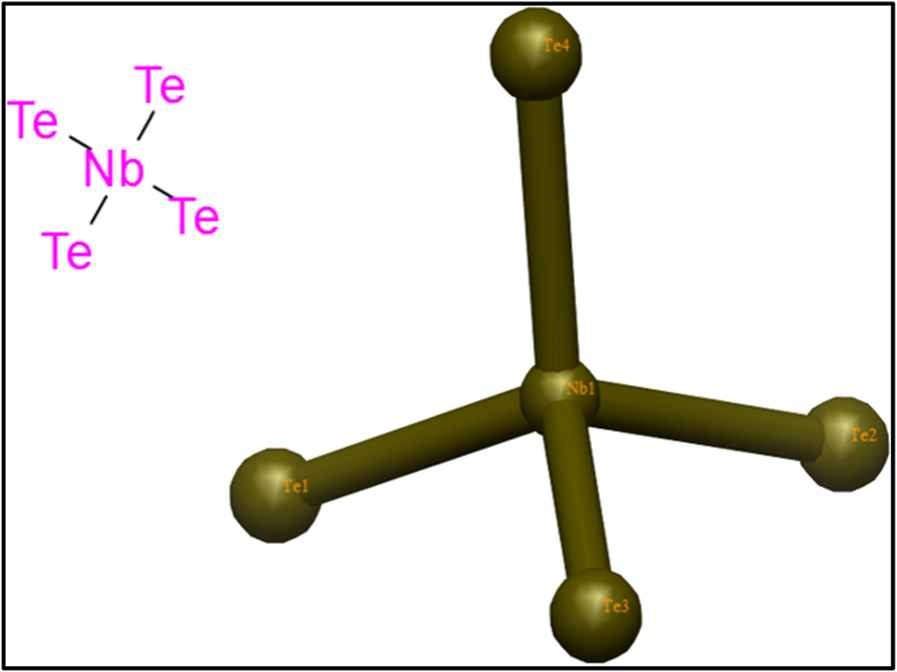
Structure of niobium-doped telluride (NbTe_4_) in 2D and 3D view

In order to understand the likely molecular as well as atomic-level events underlying antimicrobial efficacy of nanoparticles, it is important to evaluate their binding interaction with possible protein targets. The enzyme targets selected in the current study belong to biosynthetic pathways that are crucial for survival and growth of bacteria. Molecular docking study was performed to evaluate the binding interaction pattern of metal-doped telluride and selenide with multiple enzyme targets belonging to *E. coli* and *S. aureus*. Best docked complexes were obtained for niobium-doped telluride (NbTe_4_) (see Fig. 10) with enoyl-[acyl-carrier-protein] reductase (FabI) and beta-ketoacyl-acyl carrier protein synthase III (FabH) of *S. aureus* and *E. coli*, respectively.

**Fig. 11:**
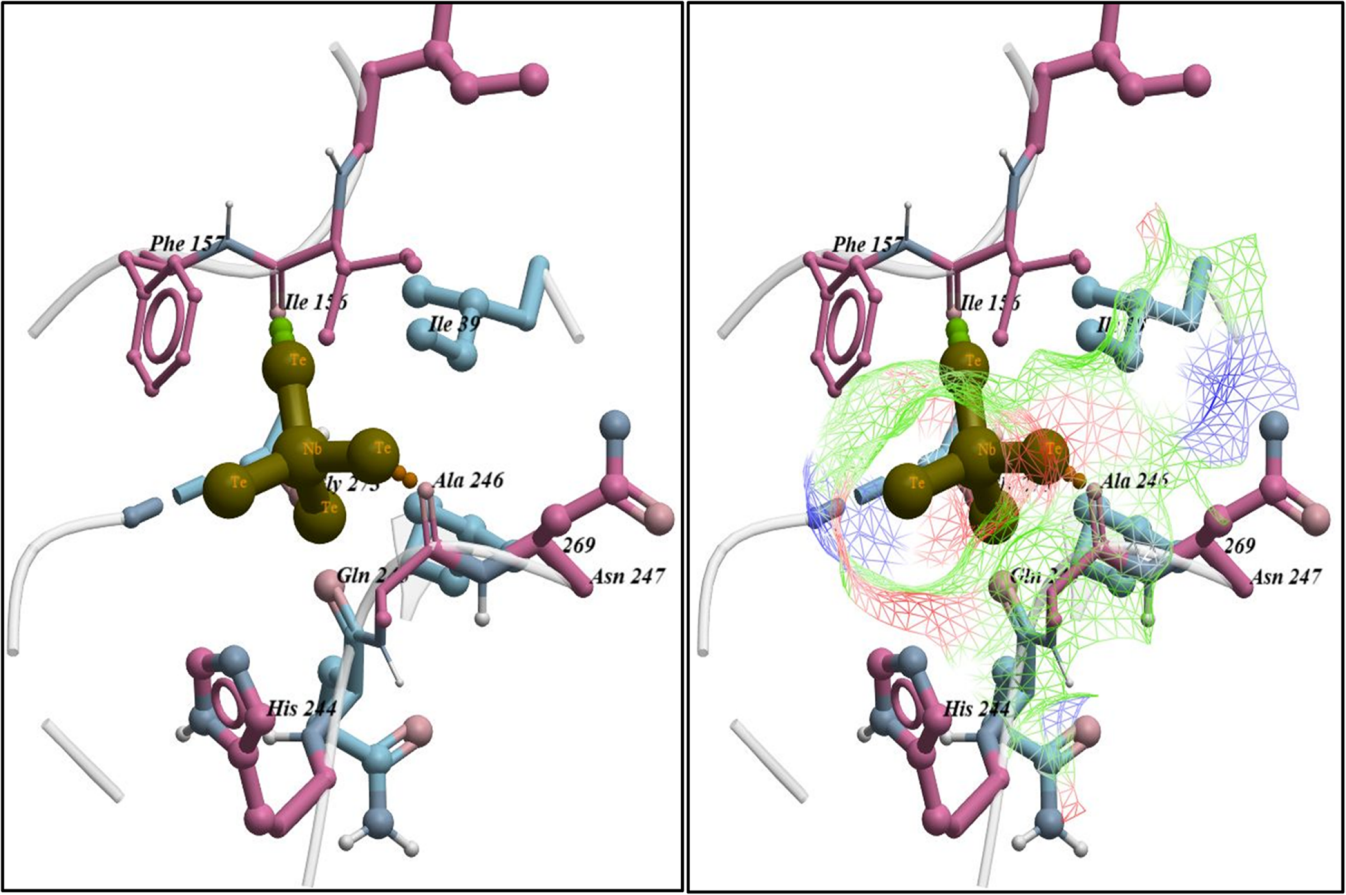
Binding interaction pattern of NbTe_4_ NPs with active site residues of beta-ketoacyl-acyl carrier protein synthase III (FabH) from *E. coli*

**Fig. 12:**
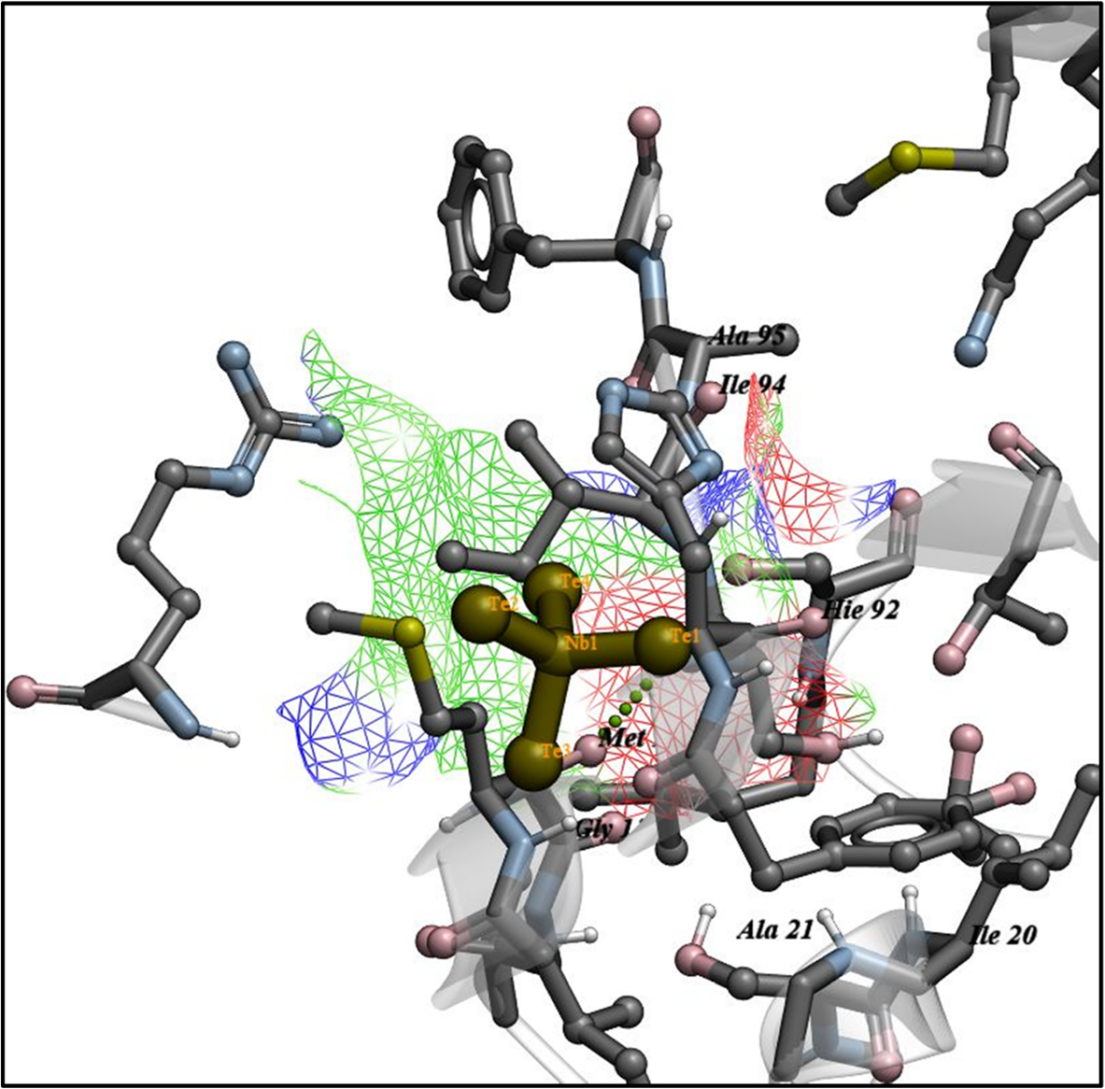
Binding interaction pattern of NbTe_4_ NPs with active site residues of enoyl-[acyl-carrier-protein] reductase (FabI) from *S. aureus*

The docking score obtained for best-docked configuration of NbTe_4_ with beta-ketoacyl-acyl carrier protein synthase III (FabH) of *E. coli* was −4.361 kcal/mol. The NbTe_4_ NPs formed H-bonding interactions with Ala246 and Ile156 with a bond distance of 1.4 Å and 1.5 Å as shown in Fig. 11. In addition, the enoyl-[acyl-carrier-protein] reductase (FabI) represents another important enzyme of the fatty acid biosynthetic pathway and its inhibition can lead to the death of bacteria. The binding score −3.829 kcal/mol obtained for docking of NbTe_4_ NPs into active pocket of FabI is attributed to H-bonding interaction with Met12 and metal contact with Gly13 as depicted in Fig. 12.

In silico findings are in good agreement with an antimicrobial activity where NbTe_4_ NPs showed the largest zones of inhibition suggesting it to be a potential inhibitor against FabH and FabI enzymes belonging to the fatty acid biosynthetic pathway. It is important to address the concerns regarding the potential toxic effect of these nanoparticles on humans and animals alike. In human beings, no toxic effects of niobium metal have been reported thus far; however, in animals, it has shown higher toxicity compared to tantalum and various other rare elements that were tested. The inhibitory effect of niobium on mouse liver enzyme succinic dehydrogenase was first noticed by Horecker et al. [87] and later investigated by Cochran and his associates [88] who ascribed the toxicity of niobium to its interference with the metal-activated enzymatic reactions. Tellurium biochemistry in the perspective of human and animal toxicology has been reviewed to a lesser extent compared to that of selenium. Although tellurium and selenium show many chemical similarities, the nutritional role of tellurium has never been reported. Moreover, minute concentrations of Te has been reported to induce chronic as well as acute toxicity in various organisms [89]. In the biological environment, Te behaves differently as it is less soluble in physiological PH and easily oxidizes to tellurite (TeO_3_^−2^), tellurate (TeO_4_^−2^), or TeO_2_ as compared to Se. Tellurium dioxide is water insoluble at biotic PH and the reduced product of tellurium, H_2_Te decomposes readily under the effect of light and air when compared to H_2_Se. These characteristics attributed to tellurium renders it a less bio-toxic element than selenium. Similar to other mammals, after the injection of tellurium salts in humans, reduction and methylation occur, which results in the formation of Te^0^ and (CH_3_)_2_Te that is eliminated from the body through breathing, urination, and sweating [90].

## Conclusions

The compounds of two classes namely selenides and tellurides of transition metals (Ta and Nb) were successfully synthesized with compositions of TaSe_3_, Nb_2_Se_3_, and TaTe_4_, NbTe_4_ through standard solid-state technique. Crystallographic and morphological evidence indicated crystallization of monoclinic selenides and tetragonal tellurides that suggests particle agglomeration tendency in the nano regime. The estimated average crystallite size (~ 10-22 nm) and d-spacings (0.31 nm) of (111) plane, and (0.34 nm) of (002) plane obtained from XRD were in accordance to HR-TEM results. The presence of all constituent elements (Ta, Nb, Se, Te, and O) in respective samples consistent with their relative atomic proportions was confirmed with EDS spectra. Transmittance and absorption peaks in FTIR and Raman spectra obtained from NPs indicated the presence of Nb-O/Te-O, TaO_6_, NbO_4_, Se-O/Ta_2_O_5_ Se-O/Nb-O structural units. Optical properties disclosed that both groups of extracted products are semiconductors with wide band gaps energies (3.82-3.99 eV) while NbTe_4_ and TaSe_3_ exhibit good catalytic potential compared to TaTe_4_ and Nb_2_Se_3_ owing to the slight differences in their structures. Besides, substantial antibacterial efficacy of telluride clusters against (G + ve) *Staphylococcus aureus* and (G –ve) *Escherichia coli* suggested that transition metal tellurides are promising bactericidal managers compared to selenide class of transition metals. Molecular docking investigation of NbTe_4_ crystals showed remarkable binding score and interaction mechanism inside the active site of targeted proteins suggesting that it could be used as a potential inhibitor of FabH and FabI enzymes and can be further probed for its inhibition characteristics.

## Data Availability

All data are fully available without restriction.

## Abbreviations

Eg: Band gap energy
EDS: Energy dispersive X-ray spectroscopy
FESEM: Field emission scanning electron microscope
FTIR: Fourier transform infrared spectroscopy
(G + ve): Gram positive
(G –ve): Gram negative
HR-TEM: High resolution transmission electron microscope
JCPDS: Joint committee on powder diffraction standards
MB: Methylene blue
nm: Nanometer
Nb: Niobium
PL: Photoluminescence
UV-Vis: Ultra-violet visible spectroscopy
Ta: Tantalum
XRD: X-ray diffraction
